# Metacognitive Illusions: A Positivity Effect in Judgments of Learning for Older but Not Younger Adults

**DOI:** 10.3390/jintelligence11030040

**Published:** 2023-02-21

**Authors:** Xiaojun Sun, Yingjie Jiang

**Affiliations:** School of Psychology, Northeast Normal University, Changchun 130024, China

**Keywords:** judgments of learning, emotional salience, metamemory, emotion, positivity, aging

## Abstract

The positivity effect for metacognitive judgments (judgments of learning, JOLs) of emotional words in recognition memory was shown in older adults, in contrast to younger adults, who typically displayed the emotional salience effect. This is compatible with the socioemotional selection theory, which suggests the presence of a positive stimulus bias in older adults’ cognitive processes. This study examined whether the positivity effect and age-related differences could be extended to a picture study to determine whether the positivity effect in older adults is robust in the metacognitive domain. Younger and older adults studied negative, positive, and neutral pictures, followed by JOLs and then a recognition test that asked participants to judge whether the picture was shown in the studying stage or not. Age-related differences were found not only in recognition memory performance for emotional pictures but also in JOLs and their accuracy. Younger adults showed an emotional salience effect for both memory performance and JOLs. Older adults’ JOLs showed a positivity effect, but their actual memory performance was influenced by emotion, and this inconsistency between metacognitive judgments and memory performance is a metacognitive illusion. These findings support the cross-material replicability of a positivity bias in older adults in the metacognitive domain and suggest that we should be cautioned about the detrimental effects of this metacognitive illusion in older adults. It illustrates an age difference in the effect of emotion on individual metacognitive monitoring ability.

## 1. Introduction

Metacognition is the ability to monitor and control memory processes (Dunlosky and Metcalfe 2008), and judgments of learning (JOLs) is often used to examine an individual’s ability to monitor memory performance (Metcalfe and Dunlosky 2008). JOLs refer to a predictive judgment of the likelihood that a participant will correctly recall the stimulus on a subsequent test immediately after learning the items. Analytic processing theory (AP) emphasizes that participants make JOLs by looking for cues that they believe will influence their memory and make JOLs based on these cues (Rhodes 2016; Tauber et al. 2019). Knowing which cues influence JOLs is crucial as people base their subsequent decisions about what and how to learn on these cues. Accurate JOLs could promote learning (Kelly and Metcalfe 2011), enabling individuals to effectively monitor, regulate, and control their mental processes (Koriat and Helstrup 2007). However, JOLs are susceptible to a variety of illusions and biases, which may cause metacognitive illusions when such individual-dependent judgment cues do not contribute to actual memory performance (Rhodes and Castel 2008), limiting their effective regulation of subsequent learning and thus impairing learning efficiency.

Numerous studies have demonstrated that emotion is a cue for individuals to make JOLs, meaning that JOLs are influenced by the emotional properties of the memory material (Fairfield et al. 2015; Hourihan and Bursey 2017; Hourihan et al. 2017; Hourihan 2020; Schmoeger et al. 2020; Undorf et al. 2018; West and Mulligan 2021; Witherby and Tauber 2018). The majority of studies that have examined the effect of emotion on JOLs have focused on younger groups and have found that younger adults tend to give higher JOLs to emotional stimuli than to neutral stimuli (Hourihan 2020; Schmoeger et al. 2020). In contrast, research on the effects of emotions on JOLs in older adults is more scarce and has not yielded consistent findings (Sanders and Berry 2021; Tauber and Dunlosky 2012; Tauber et al. 2017). An important issue in the field of metacognitive aging research is determining whether younger and older adults use similar cues when forming JOLs, as the validity of the specific types of cues individuals rely on when forming JOLs can affect metacognitive accuracy (Castel et al. 2016; Koriat 1997). Tauber and Dunlosky (2012) first explored the effects of aging on learning monitoring of emotional material and found that older adults were insensitive to metacognitive monitoring of positive words. That is, older adults’ JOLs did not differ between positive and neutral words, but this result was not replicated in subsequent research.

According to socioemotional selectivity theory, as people age, the goal of focusing on emotional well-being and emotionally meaningful aspects of life becomes prioritized (Mather and Carstensen 2005; Mather and Knight 2005). That is, younger adults tend to focus equally or more on negative and positive experiences, while older adults focus more on positive experiences (Mather and Carstensen 2005; Tauber and Dunlosky 2012). This leads to a “positivity effect (PE)” in older adults’ cognitive processes that prefers positive stimuli (Reed and Carstensen 2012), and this positivity bias may be an important cue in their JOLs. This positivity effect in older adults should also be reflected in metacognitive judgments: that is, older adults should have higher JOLs for positive stimuli than for negative and neutral stimuli (Sanders and Berry 2021; Witherby et al. 2021). Sanders and Berry (2021) found PEs for older adults’ JOLs by having older adults study negative, positive, and neutral words, making JOLs after learning each word, and then being asked to take a recognition memory test. The results found that older adults made higher JOLs for positive words than for both negative and neutral words, while there was no significant difference in JOLs for negative and neutral words. This is consistent with the socioemotional selectivity theory indicating that individuals have age-related differences in emotional bias, suggesting that this positivity bias may be a cue for older adults to make JOLs.

Notably, much of the evidence in the memory domain supporting an age-related positivity bias is based on studies of emotional images (Tauber and Dunlosky 2012). In contrast to words, pictures are more able to salience the emotional properties of stimuli. This is because pictures are more complex than words, can provide more information and more emotional detail, and the neural responses to viewing pictures tend to be more pronounced than words (Tauber et al. 2017). However, we cannot be sure whether older adults’ metamemory can show an age-related positivity bias when the learning material is emotional pictures.

Tauber et al. (2017) asked older adults to study positive and neutral pictures and then make JOLs and complete memory tests. The results found that older adults’ JOLs for positive pictures were significantly higher than those for neutral pictures for both the free recall and the recognition tasks. However, the lack of negative pictures in this study’s memory material and the relative composition of study items across emotional categories may also affect individuals’ metamemory (Zimmerman and Kelley 2010), so we cannot directly compare the results with other studies. It is, therefore, uncertain whether the phenomenon of higher positive picture JOLs than neutral picture JOLs in older adults is an emotional salience effect due to pictures that better highlight aspects of emotional stimuli (Tauber et al. 2017) or a PE specific to older adults according to socioemotional selectivity theory (Sanders and Berry 2021).

This paper aims to further clarify the role played by emotion in the metacognition of younger and older adults and to fill the gaps in previous studies. The present study investigated the effect of the interaction of emotion and age on JOLs and its accuracy when participants studied positive, negative, and neutral pictures after making JOLs in a recognition task in which the learning material also contained negative pictures. If the emotional salience effect of JOLs in older adults is the same as in younger adults because pictures are more pronounced for emotional stimulus characteristics (Tauber et al. 2017), then the emotionally salient effect in older adults should also be observed in the memory task containing negative pictures in the present study, i.e., JOLs for emotional pictures would be higher than JOLs for neutral pictures in line with younger adults. Moreover, Tauber et al. (2017) selected only positive and neutral pictures and lacked negative pictures, so the phenomenon of positive JOLs higher than neutral JOLs may have different meanings in different age groups. For younger adults, it means an emotional salience effect, while for older adults, it may indicate a PE in socioemotional selectivity theory (Sanders and Berry 2021). In the recognition task, then, we predicted that the results of the present study should be consistent with those of Sanders and Berry (2021). That is, younger adults’ JOLs would show an emotional salience effect (emotional JOLs would be higher than neutral JOLs), whereas older adults would reflect the PE, that is, positive JOLs would be higher than negative JOLs and neutral JOLs.

In summary, the main goal of the current study was to investigate whether an emotional salience effect or a PE occurs when older adults make JOLs of emotional pictures compared to younger adults and whether this effect is accurate. Exploring these questions will help to reveal the role that emotion and aging play in memory performance prediction in individual memory and metamemory and help to understand the mechanisms of metacognitive monitoring and the factors that limit its accuracy, which is considered essential for exploring practical interventions to calibrate learning performance, reduce bias, and optimize learning efficiency (Yang et al. 2021).

## 2. Materials and Methods

### 2.1. Participants

The sample size was chosen in the light of an a priori power analysis using G*Power 3.1 (Faul et al. 2007) based on previous studies (Sanders and Berry 2021; Tauber and Dunlosky 2012; Tauber et al. 2017) that estimated that at least 18 participants were required to obtain effect size (*d* = .33) with the power of 0.80. Thirty younger adults (15 males and 15 females, *M* = 21.55, *SD* = 1.99) were recruited from the school, and thirty-four older adults (17 males and 17 females, *M* = 64.66, *SD* = 3.97) were recruited from the community. Two older adults and one younger adult were dropped because of inattentive attitude or computer program error, and sixty-one valid participants were retained. The participants were all right-handed, with normal or corrected visual acuity and no mental or psychological disorders. The participants were paid after completing the experiment.

### 2.2. Materials

Two hundred and forty pictures were selected from the Chinese Affective Picture System (CAPS), including 80 positive, 80 negative, and 80 neutral pictures (see Table 1). Half of these pictures were randomly selected for the encoding phase (120 pictures: 40 each of positive, negative, and neutral pictures), and the other half were presented as new pictures for the recognition phase (120 pictures: 40 each of positive, negative, and neutral pictures). All picture sets (positive, negative, and neutral pictures) were significantly different on mean valence (all *p* < .001). The sets of positive and negative pictures did not differ from one another on mean arousal (*p* = .712) but were significantly higher in arousal than the neutral pictures (*p* < .001). The total picture set was divided into pictures presented in the encoding phase and new pictures presented in the recognition phase to be balanced between participants, i.e., half of the picture set presented in the encoding phase of the participants corresponded to the new picture set presented in the recognition phase of the other half of the participants.

### 2.3. Task Design and Procedure

#### 2.3.1. Task Design

A mixed experimental design was used for this experiment: 2 (age group: younger adults vs. older adults) × 3 (picture valence: negative vs. positive vs. neutral), with age group (younger adults/older adults) as the between-subjects variable and picture valence (negative/positive/neutral) as the within-subjects variable.

#### 2.3.2. Procedure

This experiment contains an encoding phase and a recognition phase. During the picture encoding phase, there were a total of 120 trials containing 40 positive, 40 negative, and 40 neutral pictures presented in random order. Each picture was presented on a computer screen for 1000 ms and was preceded by a 500 ms fixation cross to ensure that the participants fixated on the center of the screen. The participants were instructed to remember the pictures. Immediately after studying each picture, a self-paced JOL was made for that picture by predicting the likelihood of remembering that picture on a future test. JOLs were made on a scale from 0 (certain the picture would not be remembered) to 100 (certain the picture would be remembered).

After the final JOLs were provided, participants completed three minutes of a distraction task before completing the recognition phase. The distraction task is a mathematical operation of subtracting the number 3 from a randomly presented three-digit number for a continuous period of 3 min, followed by a verbal report of the result. In the recognition phase, the test consisted of 240 trials (120 studied and 120 new), presented in random order. Each picture was presented on the computer screen until the response was made by the participant and was preceded by a 500 ms fixation cross. The picture remained visible until one of the response keys with the keypress labels for “old” and “new” was pressed.

### 2.4. Statistical Analyses

We used IBM SPSS 26.0 to conduct two-way mixed ANCOVAs with age group (younger adults, older adults) as between-subjects and picture valence (negative, positive, neutral) as within-subjects. JOLs magnitude, picture corrected recognition scores, and JOLs accuracy were dependent variables. Corrected recognition scores were calculated by subtracting the proportion of pictures that participants reported as viewed but were novel (FAs) from the proportion of correctly identified previously viewed pictures (HITs) (Charles et al. 2003; Massol et al. 2020). These were followed by follow-up tests. All the results in the text and figures of this study are expressed as percentages.

## 3. Results

### 3.1. JOLs Magnitude

**Figure 1:**
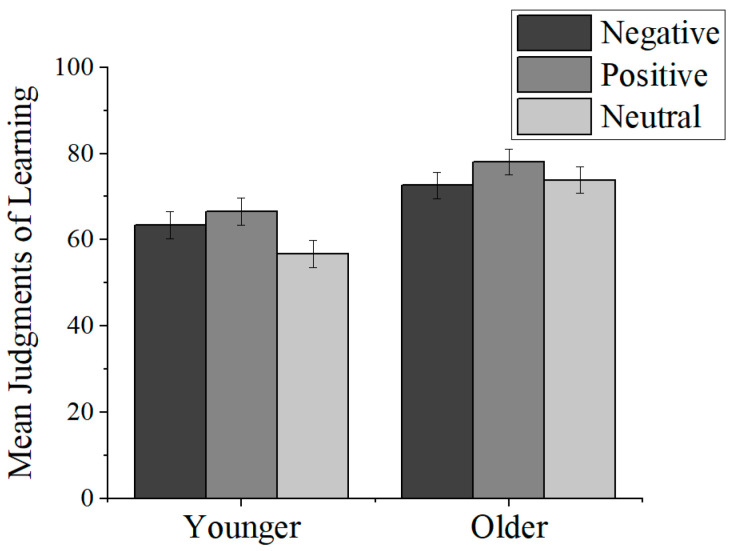
Mean judgments of learning (JOLs) responses by valence for younger and older adults (with standard error bars).

As evident from Figure 1, both younger and older adults’ JOLs were sensitive to positive valence, with the magnitude of the predictions being higher for positive pictures compared with neutral pictures. In contrast to older adults, however, younger adults’ JOLs were also higher for negative pictures than for neutral ones. These observations were supported by a 2 (age group: younger adults, older adults) × 3 (picture valence: negative, positive, neutral) mixed-factor analysis of variance (ANOVA). A significant main effect of age group, *F*(1, 59) = 9.00, *p* = .004, η_p_^2^ = .132, indicated that older adults’ JOLs were significantly higher than younger adults’ JOLs. The main effect of picture valence, *F*(2, 118) = 44.93, *p* < .001, η_p_^2^ = .608, was qualified by a significant picture valence by age group interaction, *F*(2, 118) = 15.57, *p* < .001, η_p_^2^ = .349. Follow-up tests indicated that for younger adults, JOLs were significantly higher for negative than neutral pictures, *t*(28) = 4.20, *p* < .001, *d* = .78, and were higher for positive than neutral pictures, *t*(28) = 8.33, *p* < .001, *d* = 1.55. Younger adults’ JOLs between positive and negative pictures did not differ, *t*(28) = 1.64, *p* = .065, *d* = .30. Thus, younger adults’ JOLs confirmed the predicted emotional salience effect. Older adults’ JOLs were significantly higher for positive pictures than neutral pictures, *t*(31) = 4.29, *p* < .001, *d* = .76, and for negative pictures, *t*(31) = 4.02, *p* = .001, *d* = .71, supporting the predicted PE. Older adults’ JOLs did not differ between neutral and negative pictures, *t*(31) = 1.34, *p* = .324, *d* = .24.

### 3.2. Recognition Memory Performance

**Figure 2:**
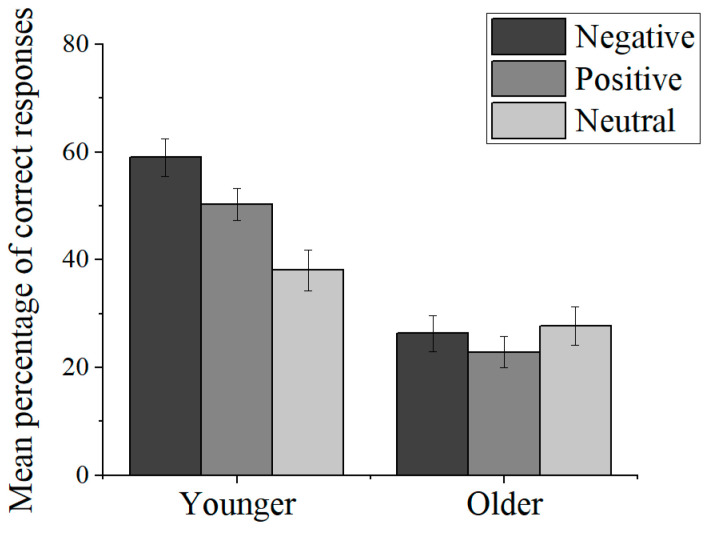
Mean percentage of correct responses (corrected recognition scores: correctly recognized items (HITs)-false alarms (FAs) for pictures) by valence for younger and older adults (with standard error bars).

As shown in Figure 2, younger adults showed superior memory for emotional pictures compared to neutral pictures. However, in contrast to younger adults, there was no emotional salience effect in the memory of older adults. These observations were supported by a 2 (age group: younger adults, older adults) × 3 (picture valence: negative, positive, neutral) mixed-factor analysis of variance (ANOVA). A significant main effect of age group, *F*(1, 59) = 32.35, *p* < .001, η_p_^2^ = .354, indicated better recognition of younger adults than older adults. The main effect of picture valence was significant, *F*(2, 118) = 11.71, *p* < .001, η_p_^2^ = .166, which was qualified by a significant valence by age group interaction, *F*(2, 118) = 16.42, *p* < .001, η_p_^2^ = .218. Follow-up tests indicated that for younger adults, recognition for negative pictures was significantly higher than for positive, *t*(28) = 4.08, *p* = .007, *d* = .76, and neutral pictures, *t*(28) = 7.12, *p* < .001, *d* = 1.32, and for positive pictures were significantly higher than for neutral pictures, *t*(28) = 4.37, *p* < .001, *d* = .81. For the older adults, recognition did not vary by valence, *F*(1, 59) = 2.05, *p* = .139, η_p_^2^ = .066.

### 3.3. JOLs Resolution Accuracy

Resolution accuracy can be expressed as the Goodman–Kruskal γ coefficient to assess the extent to which participants’ JOLs at the trial level can distinguish between correctly versus incorrectly recognized items (Nelson 1984). The γ correlation could not be calculated in the absence of variance, so one older adult who gave the same JOLs in all trials and another who answered correctly to all positive pictures in the test trials were excluded. The resolution did not significantly vary as a function of age group, *F*(1, 57) = .29, *p* = .590, η_p_^2^ = .005. The main effect of valence was nonsignificant, *F*(2, 114) = .84, *p* = .436, η_p_^2^ = .014. The Age group × Valence interaction was nonsignificant, *F*(2, 114) = 1.75, *p* = .180, η_p_^2^ = .030. One-sample *t*-tests (comparing each group means against zero) demonstrated that younger adults’ γ correlations overall (*M* = .15, *SE* = .04, *p* < .001) and for neutral pictures (*M* = .19, *SE* = .06, *p* = .005) were significantly greater than zero, and for negative (*M* = .12, *SE* = .07, *p* = .097) and positive pictures (*M* = .13, *SE* = .07, *p* = .080) were not greater than zero. For older adults’ γ correlations for overall (*M* = .11, *SE* = .04, *p* = .016) and negative pictures (*M* = .22, *SE* = .06, *p* = .001) were significantly greater than zero, for positive (*M* = .04, *SE* = .10, *p* = .665) and neutral pictures (*M* = .06, *SE* = .07, *p* = .371) were not greater than zero.

However, researchers have demonstrated that the use of γ to reflect resolution is biased and may lead to misleading conclusions. These problems can be avoided by using measures based on signal detection theory. The *d*_a_ has a larger magnitude of variation and a more sensitive metric, which is more suitable for measuring metacognitive accuracy than γ (Benjamin and Diaz 2008; Masson and Rotello 2009). Therefore, we further analyzed the resolution by *d*_a_. Data from fifteen participants were not included in the analysis because they failed to meet the prerequisites for *d*_a_, and data from forty-five participants were analyzed. The resolution did not significantly vary as a function of age group, *F*(1, 43) = 2.78, *p* = .103, η_p_^2^ = .061. The main effect of valence was nonsignificant, *F*(2, 86) = 2.23, *p* = .120, η_p_^2^ = .096. The Age group × Valence interaction was nonsignificant, *F*(2, 86) = 1.75, *p* = .770, η_p_^2^ = .012.

## 4. Discussion

The younger adults in the current study showed the emotional salience effect of higher JOLs for emotional information than for neutral information, which replicated the results of previous studies (Hourihan and Bursey 2017; Hourihan 2020; Sanders and Berry 2021). For younger people, this may be because they hold the belief that emotional information is easier to remember than neutral information, consciously and strategically given higher JOLs for emotional pictures (Hourihan 2020). Alternatively, it may be due to the attention-attracting properties of emotional material during encoding or the emotional responses it evokes being noticed by younger people as a cue for JOLs (Zimmerman and Kelley 2010). The JOLs of older adults found the PE, i.e., the phenomenon that older adults’ JOLs for positive pictures were significantly higher than their JOLs for negative and neutral pictures, while there was no significant difference between JOLs for negative and neutral pictures. This is consistent with the research findings of Sanders and Berry (2021) that there was a bias for positive information over negative information in older adults’ lives (Reed and Carstensen 2012) and that this positive bias was an important cue when making JOLs in the context of recognition tests. Even in picture materials that are more salient to stimulus characteristics, the subjective state of developing a motivational orientation toward positive information remained the main cue for older adults to make JOLs, further demonstrating the replicability of older adults’ positive bias in the metacognitive domain and corroborating that older adults’ positive preferences to preferentially focus on well-being and emotionally meaningful goals in life are also reflected in metacognitive judgments.

The present study observed PE results in older adults’ JOLs that differed from the insensitivity of older adults’ JOLs to metacognitive monitoring of positive words (Tauber and Dunlosky 2012). The reason for this phenomenon may be that words are not sufficient to elicit positive aspects of stimuli, whereas emotional pictures may provide older adults with more arousing emotional cues, allowing them to use bias in cognitive processes towards positive stimuli as a cue for JOLs (Reed and Carstensen 2012), thus showing a different PE from the emotional salience effect in younger adults. The evidence in the memory domain supporting age-related positivity bias is mostly based on examining emotional pictures (Tauber and Dunlosky 2012), and it is possible that the PE in the metamemory domain among older adults JOLs is also more easily observed in picture material, which of course needs to be confirmed by subsequent studies. It is also noteworthy that the free recall task was taken in the Tauber and Dunlosky (2012), our and another study supporting PE in older adults JOLs selected both a recognition task (Sanders and Berry 2021). This suggests the existence of boundary conditions for PE in older adults’ metamemory, potentially limited by the type of memory task (Nomi et al. 2013; Zimmerman and Kelley 2010). Future research could explore the boundary conditions of the PE in older adults’ JOLs, with a focus on other types of item memory and associative memory, to clarify whether different memory task types are responsible for whether older adults’ metamemory can show PE.

According to Koriat’s cue utilization framework, people’s monitoring of their ability to remember emotional information might depend on two types of information. The first is theory-based analytical reasoning: individuals use the inherent emotional properties of the learned material as internal cues and have formed the belief that emotional information is easier to remember than neutral information. The second is empirically or unanalytically based heuristics: the attention-attracting nature of emotional material or the emotional responses during encoding may be perceived by individuals as markers of whether response memory can be successful (Koriat 1997; Zimmerman and Kelley 2010). This analysis of the two different potential effects of JOLs is known as the two-factor theory of JOLs (Witherby et al. 2021), which can be briefly described as the different contributions of beliefs and experiences to JOLs. The first is based on beliefs in an explicit, conscious, analytical way, whereas the second is based on experience in an unconscious, non-analytical way. In addition to beliefs, immediate experiences during learning, such as fluency in processing, can also influence JOLs. Individuals will have higher JOLs for emotional stimuli than neutral stimuli if they are processed more fluently than neutral stimuli (Schaper et al. 2019; Witherby et al. 2021).

Any cue can influence JOLs through these two non-exclusive mechanisms (Witherby et al. 2021), and the effect of emotion on JOLs in younger and older adults is no exception. In our study, we found that younger adults’ JOLs showed emotional salience effects, and older adults’ JOLs showed positive effects. The analysis from the perspective of beliefs may be that younger and older adults hold different beliefs about the effect of emotion on memory, with younger adults forming the belief that emotional information is easier to remember than neutral information, whereas older adults form the belief that positive information is easier to remember than negative and neutral information. Furthermore, research has shown that the JOLs of both older and younger adults are influenced by the effect of processing fluency and that the effect of fluency is similar for both age groups, with more fluidly processed items corresponding to higher JOLs (Castel et al. 2016; Emanuel Robinson et al. 2006; Hertzog and Dunlosky 2011). From this perspective, the results of the current study may indicate that younger adults have higher processing fluency for emotional pictures than for neutral pictures and that older adults have higher processing fluency for positive pictures than for negative and neutral pictures. It is worth noting that the two pathways, belief and experience, are not completely exclusive (Rhodes 2016), so besides emotional attributes directly influencing JOLs through beliefs or experiences, it is also possible that experiences indirectly influence JOLs by influencing beliefs (Mueller and Dunlosky 2017), or that beliefs influence experiences by indirectly influence JOLs by influencing experience (Witherby et al. 2021). Unfortunately, the current study could not clarify the relative contribution of beliefs and experience pathways in younger and older JOLs and whether this contribution differs with age. Future studies could use sophisticated experimental designs and cognitive neuroscience to clarify the relative contribution of experience and beliefs.

Although the focus of the current study was to explore the effects of emotion and aging on individual metamemory, the effect of emotion on memory across age groups is also of great research interest, and memory results also were analyzed. The recognition memory results appeared to segregate across age groups, with older adults’ recognition memory not being influenced by emotion, replicating the results of previous studies (Sanders and Berry 2021). Older adults did not show PE in recognition memory, which might be due to the cognitive resources taken up by the additional JOLs task required after studying the pictures, and this reduction in cognitive resources would eliminate the PE in older adults’ memory, whereas emotional memory is not affected in younger adults (Mather and Knight 2005). Similarly, processing limitations might also account for the absence of PE in older adults’ recognition performance (Reed et al. 2014). The present experiments required participants to make JOLs after studying restricted individuals’ information processing, which might interfere with their emotion-based processing preferences and goals (Sanders and Berry 2021). Older adults were more influenced by processing constraints and no longer showed a preference for positive stimuli when information processing was constrained but processed positive and negative stimuli at an equal level. In contrast, for younger adults, there was a greater bias toward preferential processing of negative information regardless of whether information processing was constrained (Reed et al. 2014).

The recognition performance of younger adults in the present study was also consistent with the above in that their emotional enhancement of memory effect was not impaired, i.e., memory for emotional information was better than for neutral information (Massol et al. 2020). This emotional enhancement of memory effect was found for a variety of emotional events such as words, pictures, and stories, and this phenomenon may occur because emotional stimuli are more likely to attract attention than neutral information, leading to an increase in individuals’ attentional resources for emotional stimuli and, in turn, better memory performance for emotional stimuli (Humphreys et al. 2010). Specifically, younger adults showed better recognition memory for positive pictures than neutral pictures and for negative pictures than positive and neutral pictures, which replicates the results of previous studies on emotional memory (Kark and Kensinger 2019). Positive stimuli are persistent and accessible compared to neutral stimuli, and the rewarding nature of learning positive material can facilitate memory, so memory performance for positive pictures is better than for neutral pictures (Williams et al. 2022). Negative stimuli had the best memory performance, suggesting that younger adults showed a negative bias in memory, and this finding is supported by the results of previous studies (Humphreys et al. 2010). Negative pictures have better memory performance than neutral and positive pictures, probably because negative stimuli have sensory specificity, recapitulation vividness, and prioritization of retrieval, gaining additional cognitive resources during encoding and provoking memory mechanisms to further consolidate memory (Williams et al. 2022). Furthermore, the amygdala is a key link to emotionally enhanced memory, and researchers have found that negative memory bias can be linked to the way sensory processes are integrated into the amygdala-centered emotional memory network, which explains why people have enhanced memory for negative events, but not a positive or neutral event (Kark and Kensinger 2019).

We also analyzed the JOLs’ accuracy, found no age differences in the resolution of emotional pictures monitoring, and monitoring resolution was unaffected by picture validity for both age groups. This replicates previous findings suggesting that even the monitoring of emotional material is largely unaffected by age (Sanders and Berry 2021; Tauber and Dunlosky 2012; Tauber et al. 2017). These results support the theoretical view of metacognitive aging that monitoring of learning is largely unaffected by age, that the resolution of monitoring tends to remain stable over a person’s lifetime, and that older adults can monitor their memory performance as effectively and accurately as younger adults (Castel et al. 2016; Hertzog and Dunlosky 2011; Tauber and Dunlosky 2012; Tauber and Witherby 2016).

It is worth noting, however, that the above metacognitive monitoring ability for emotional information is given based on resolution. As an alternative to the relative accuracy represented by resolution, whether metacognitive judgments and actual performance follow the same pattern across conditions may also reflect an individual’s metacognitive monitoring ability (Koriat 2007; Undorf et al. 2022). If an individual’s metacognitive judgments and cognitive performance are consistent, then it is accurate. Otherwise, it indicates a metacognitive illusion (Undorf et al. 2022). From this perspective, younger adults’ JOLs and actual performance were both higher for emotional pictures than for neutral pictures, i.e., younger adults’ metacognitive judgments and actual performance showed the same pattern across conditions. This suggests that younger adults’ JOLs are accurate, and there is no metacognitive illusion. The situation is different for older adults, who believe that positive pictures are best remembered, but whose actual performance is not influenced by emotion. This inconsistency between metacognitive judgments and actual performance in older adults suggests that PE in older adults’ JOLs is a metacognitive illusion. Metacognitive illusions might lead to biased and ineffective learning behavior (Berlamont et al. 2022), limiting their effective modulation of subsequent learning and impairing learning efficiency (Tauber and Dunlosky 2012). The results of this finding provided insight that we should pay more attention to the different roles that age factors play in individuals’ metacognitive monitoring of emotional information. For example, we should pay attention to the metacognitive illusions induced by positive emotional attributes in older adults to avoid the detrimental effects of metacognitive illusions. Improving the accuracy of metacognitive processes can improve older adults’ quality of life (Tauber and Witherby 2016), and effective use of metacognitive monitoring can help older adults compensate for memory decline through selective, compensatory learning efforts (Hertzog and Dunlosky 2011). Because of the causal relationship between metamemory monitoring, control, and memory, correcting metamemory illusions holds promise for de-biasing learning behaviors and ultimately improving memory (Schaper et al. 2022). Future research could explore in greater depth the effect of emotion on metacognitive monitoring accuracy in different age groups, which is crucial for understanding the factors that limit monitoring accuracy and how to improve individual monitoring accuracy.

## 5. Conclusions

In conclusion, this study provides evidence that the positivity bias in older adults functions in the metamemory domain and demonstrates the replicability and robustness of this metamemory PE in recognition tests. It is shown that age-related differences are reflected in both emotional memory and metamemory. Younger adults’ memory performance and JOLs showed emotional salience effects. Older adults showed a PE, but their actual memory performance was not affected by emotion, suggesting that this PE is a metacognitive illusion. The current findings emphasize that we should pay particular attention to the different roles that age plays in individuals’ metacognitive monitoring of emotional information. This allows individuals to more effectively avoid the detrimental effects of metacognitive illusions in memory and learning, enabling them to effectively monitor, regulate and control their cognitive processes and thus improve their learning efficiency.

## Figures and Tables

**Table 1:** Mean valence and arousal ratings of negative, positive, and neutral picture pools.

		Negative	Positive	Neutral
Valence	*M*	2.14	7.33	5.58
	*SD*	0.31	0.25	0.56
	Range	1.26–2.52	7.00–7.85	4.30–6.96
Arousal	*M*	5.88	5.85	3.91
	*SD*	0.65	0.53	0.60
	Range	4.54–6.89	5.15–7.20	2.59–5.85

## Data Availability

The data presented in this study are available from the corresponding author upon request at jiangyj993@nenu.edu.cn. The data is not shown due to participant privacy.

## References

[B1-jintelligence-11-00040] Benjamin Aaron S., Diaz Michael (2008). Measurement of relative metamnemonic accuracy. Handbook of Memory and Metamemory.

[B2-jintelligence-11-00040] Berlamont Liesbet, Sels Laura, Ickes William, Ceulemans Eva, Hinnekens Céline, Verhofstadt Lesley (2022). Associations between affect and empathic accuracy during conflict interactions in couples. Journal of Social and Personal Relationships.

[B3-jintelligence-11-00040] Castel Alan D., Middlebrooks Catherine D., McGillivray Shannon (2016). Monitoring memory in old age: Impaired, spared, and aware. The Oxford Handbook of Metamemory.

[B4-jintelligence-11-00040] Charles Susan Turk, Mather Mara, Carstensen Laura L. (2003). Aging and emotional memory: The forgettable nature of negative images for older adults. The Journal of Experimental Psychology: General.

[B5-jintelligence-11-00040] Dunlosky John, Metcalfe Janet (2008). Metacognition.

[B6-jintelligence-11-00040] Emanuel Robinson A., Hertzog Christopher, Dunlosky John (2006). Aging, encoding fluency, and metacognitive monitoring. Aging, Neuropsychology, and Cognition.

[B7-jintelligence-11-00040] Fairfield Beth, Mammarella Nicola, Palumbo Rocco, Di Domenico Alberto (2015). Emotional Meta-Memories: A Review. Brain Sciences.

[B8-jintelligence-11-00040] Faul Franz, Erdfelder Edgar, Lang Albert-Georg, Buchner Axel (2007). G*Power 3: A flexible statistical power analysis program for the social, behavioral, and biomedical sciences. Behavior Research Methods.

[B9-jintelligence-11-00040] Hertzog Christopher, Dunlosky John (2011). Metacognition in Later Adulthood: Spared Monitoring Can Benefit Older Adults’ Self-regulation. Current Directions in Psychological Science.

[B10-jintelligence-11-00040] Hourihan Kathleen L. (2020). Misleading emotions: Judgments of learning overestimate recognition of negative and positive emotional images. Cognition & Emotion.

[B11-jintelligence-11-00040] Hourihan Kathleen L., Bursey Elliott (2017). A misleading feeling of happiness: Metamemory for positive emotional and neutral pictures. Memory.

[B12-jintelligence-11-00040] Hourihan Kathleen L., Fraundorf Scott H., Benjamin Aaron S. (2017). The influences of valence and arousal on judgments of learning and on recall. Memory & Cognition.

[B13-jintelligence-11-00040] Humphreys Louise, Underwood Geoffrey, Chapman Peter (2010). Enhanced memory for emotional pictures: A product of increased attention to affective stimuli?. European Journal of Cognitive Psychology.

[B14-jintelligence-11-00040] Kark Sarah M., Kensinger Elizabeth A. (2019). Post-Encoding Amygdala-Visuosensory Coupling Is Associated with Negative Memory Bias in Healthy Young Adults. Journal of Neuroscience.

[B15-jintelligence-11-00040] Kelly Karen J., Metcalfe Janet (2011). Metacognition of Emotional Face Recognition. Emotion.

[B16-jintelligence-11-00040] Koriat Asher (1997). Monitoring One’s Own Knowledge during Study: A Cue-Utilization Approach to Judgments of Learning. Journal of Experimental Psychology: General.

[B17-jintelligence-11-00040] Koriat Asher (2007). Metacognition and Consciousness. The Cambridge Handbook of Consciousness.

[B18-jintelligence-11-00040] Koriat Asher, Helstrup Tore (2007). Metacognitive aspects of memory. Everyday Memory.

[B19-jintelligence-11-00040] Massol Sarah, Vantaggio Sophie, Chainay Hanna (2020). Emotional modulation of episodic memory in school-age children and adults: Emotional items and their associated contextual details. Journal of Experimental Psychology: General.

[B20-jintelligence-11-00040] Masson Michael E. J., Rotello Caren M. (2009). Sources of bias in the Goodman–Kruskal gamma coefficient measure of association: Implications for studies of metacognitive processes. Journal of Experimental Psychology: Learning, Memory, and Cognition.

[B21-jintelligence-11-00040] Mather Mara, Carstensen Laura L. (2005). Aging and motivated cognition: The positivity effect in attention and memory. Trends in Cognitive Sciences.

[B22-jintelligence-11-00040] Mather Mara, Knight Marisa (2005). Goal-directed memory: The role of cognitive control in older adults’ emotional memory. Psychology and Aging.

[B23-jintelligence-11-00040] Metcalfe Janet, Dunlosky John, Roediger Henry L. (2008). Metamemory. Learning and Memory: A Comprehensive Reference.

[B24-jintelligence-11-00040] Mueller Michael L., Dunlosky John (2017). How beliefs can impact judgments of learning: Evaluating analytic processing theory with beliefs about fluency. Journal of Memory and Language.

[B25-jintelligence-11-00040] Nelson Thomas O. (1984). A comparison of current measures of the accuracy of feeling-of-knowing predictions. Psychological Bulletin.

[B26-jintelligence-11-00040] Nomi Jason S., Rhodes Matthew G., Cleary Anne M. (2013). Emotional facial expressions differentially influence predictions and performance for face recognition. Cognition & Emotion.

[B27-jintelligence-11-00040] Reed Andrew E., Carstensen Laura L. (2012). The theory behind the age-related positivity effect. Frontiers in Psychology.

[B28-jintelligence-11-00040] Reed Andrew E., Chan Larry, Mikels Joseph A. (2014). Meta-analysis of the age-related positivity effect: Age differences in preferences for positive over negative information. Psychology and Aging.

[B29-jintelligence-11-00040] Rhodes Matthew G. (2016). Judgments of learning: Methods, data, and theory. The Oxford Handbook of Metamemory.

[B30-jintelligence-11-00040] Rhodes Matthew G., Castel Alan D. (2008). Memory Predictions Are Influenced by Perceptual Information: Evidence for Metacognitive Illusions. Journal of Experimental Psychology-General.

[B31-jintelligence-11-00040] Sanders Edie C., Berry Jana M. (2021). Evidence for an Age-Related Positivity Effect in Metacognitive Judgments. The Journals of Gerontology Series B, Psychological Sciences and Social Sciences.

[B32-jintelligence-11-00040] Schaper Marie Luisa, Kuhlmann Beatrice G., Bayen Ute J. (2019). Metacognitive expectancy effects in source monitoring: Beliefs, in-the-moment experiences, or both?. Journal of Memory and Language.

[B33-jintelligence-11-00040] Schaper Marie Luisa, Bayen Ute J., Hey Carolin V. (2022). Remedying the Metamemory Expectancy Illusion in Source Monitoring: Are there Effects on Restudy Choices and Source Memory?. Metacognition and Learning.

[B34-jintelligence-11-00040] Schmoeger Michaela, Deckert Matthias, Loos Eva, Willinger Ulrike (2020). How influenceable is our metamemory for pictorial material? The impact of framing and emotionality on metamemory judgments. Cognition.

[B37-jintelligence-11-00040] Tauber Sarah K., Witherby Amber E., Dunlosky John (2019). Beliefs about memory decline in aging do not impact judgments of learning (JOLs): A challenge for belief-based explanations of JOLs. Memory & Cognition.

[B35-jintelligence-11-00040] Tauber Sarah K., Witherby Amber E. (2016). Metacognition in Older Adulthood. Encyclopedia of Geropsychology.

[B36-jintelligence-11-00040] Tauber Sarah K., Dunlosky John (2012). Can older adults accurately judge their learning of emotional information?. Psychology and Aging.

[B38-jintelligence-11-00040] Tauber Sarah K., Dunlosky John, Urry Heather L., Opitz Philipp C. (2017). The effects of emotion on younger and older adults’ monitoring of learning. Aging, Neuropsychology, and Cognition.

[B39-jintelligence-11-00040] Undorf Monika, Sollner Anke, Broder Arndt (2018). Simultaneous utilization of multiple cues in judgments of learning. Memory & Cognition.

[B40-jintelligence-11-00040] Undorf Monika, Navarro-Báez Sofia, Zimdahl Malte F. (2022). Metacognitive illusions. Cognitive Illusions: Intriguing Phenomena in Thinking, Judgment, and Memory.

[B41-jintelligence-11-00040] West John T., Mulligan Neil W. (2021). Investigating the replicability and boundary conditions of the mnemonic advantage for disgust. Cognition & Emotion.

[B42-jintelligence-11-00040] Williams Samantha E., Ford Jaclyn H., Kensinger Elizabeth A. (2022). The power of negative and positive episodic memories. Cognitive, Affective, & Behavioral Neuroscience.

[B43-jintelligence-11-00040] Witherby Amber E., Tauber Sarah K. (2018). Monitoring of learning for emotional faces: How do fine-grained categories of emotion influence participants’ judgments of learning and beliefs about memory?. Cognition & Emotion.

[B44-jintelligence-11-00040] Witherby Amber E., Tauber Sarah K., Dunlosky John (2021). Why do emotional stimuli influence judgments of learning? Theory, evidence, and future directions. Trends and Prospects in Metacognition Research across the Life Span.

[B45-jintelligence-11-00040] Yang Chunliang, Yu Rongjun, Hu Xiao, Luo Liang, Huang Tina S. T., Shanks David R. (2021). How to assess the contributions of processing fluency and beliefs to the formation of judgments of learning: Methods and pitfalls. Metacognition and Learning.

[B46-jintelligence-11-00040] Zimmerman Carissa A., Kelley Colleen M. (2010). “I’ll remember this!” Effects of emotionality on memory predictions versus memory performance. Journal of Memory and Language.

